# Real time curriculum map for internal medicine residency

**DOI:** 10.1186/1472-6920-7-42

**Published:** 2007-11-07

**Authors:** Roger Y Wong, J Mark Roberts

**Affiliations:** 1 Postgraduate Medical Education, Department of Medicine, University of British Columbia, Vancouver, Canada

## Abstract

**Background:**

To manage the voluminous formal curriculum content in a limited amount of structured teaching time, we describe the development and evaluation of a curriculum map for academic half days (AHD) in a core internal medicine residency program.

**Methods:**

We created a 3-year cyclical curriculum map (an educational tool combining the content, methodology and timetabling of structured teaching), comprising a matrix of topics under various specialties/themes and corresponding AHD hours. All topics were cross-matched against the ACP-ASIM in-training examination, and all hours were colour coded based on the categories of core competencies. Residents regularly updated the map on a real time basis.

**Results:**

There were 208 topics covered in 283 AHD hours. All topics represented core competencies with minimal duplication (78% covered once in 3 years). Only 42 hours (15%) involved non-didactic teaching, which increased after implementation of the map (18–19 hours/year versus baseline 5 hours/year). Most AHD hours (78%) focused on medical expert competencies. Resident satisfaction (90% response) was high throughout (range 3.64 ± 0.21, 3.84 ± 0.14 out of 4), which improved after 1 year but returned to baseline after 2 years.

**Conclusion:**

We developed and implemented an internal medicine curriculum map based on real time resident input, with minimal topic duplication and high resident satisfaction. The map provided an opportunity to balance didactic versus non-didactic teaching, and teaching on medical versus non medical expert topics.

## Background

Although accreditation bodies in North America have outlined well-established, multifaceted goals (overall direction of the education program), objectives (educationally useful elements that are rotation specific) and competencies (important observable knowledge, skills and attitudes) for residency training in internal medicine, the operational delivery of formal curriculum has been challenging for many postgraduate programs. Not only is there a tremendous and growing amount of academic content to cover during residency, but programs are also expected to teach residents all 6 general core competencies mandated by the Accreditation Council for Graduate Medical Education (ACGME) in the United States [1], or all 7 roles in the CanMEDS framework mandated by the Royal College of Physicians and Surgeons (RCPSC) in Canada [2]. All of these need to be achieved within a finite training period [3], and without extending training hours beyond the resident work hour restriction guidelines [4]. In some residency programs, formal curriculum is delivered through structured teaching opportunities such as academic half days (AHD) or full days. While these academic days may be mandatory, no standardized planning tool exists. Detailed educational planning is needed to achieve the largest possible impact, so that important topics are not missed and repetition is minimized. It is also unclear if the topics taught are actually pertinent to assessment at public internal medicine examinations, such as the American College of Physician – American Society of Internal Medicine (ACP-ASIM) in-training examination. The lack of standard methodology to manage the academic content within a busy curriculum, together with uncertainty if the content covered is actually relevant, present a double challenge for residency program directors and medical educators. Such challenge has been expressed and shared among program directors at national and international meetings.

Earlier educational studies have described the potential value of creating curriculum maps, mostly in the traditional K-12 school system [5]. A curriculum map is an educational tool that combines what is taught (the academic content), how it is taught (the teaching resources and opportunities), when it is taught (the timetable), and the measures used to assess if teaching has successfully occurred [6]. Like a road map to knowledge, a curriculum map makes the formal curriculum more transparent to all stakeholders and clarifies the relationships and links [6]. The process of establishing a map is, however, time and effort intensive. In health education, curriculum mapping has been used to help teach evidence based medicine [7], geriatrics [8], clinical nutrition [9], and computer-based virtual medical courses [10].

Curriculum mapping is a dynamic process, requiring ongoing (real time) feedback and needs assessment from the learners [11]. Ongoing feedback is commonly used in web based learning [12], which can be assessed using structured questionnaires [13,14] or focus groups [15]. We elected to have a designated group of residents from all training years to provide feedback, as this motivates residents to actively participate in their own education.

We recently developed a real time curriculum map specifically designed for internal medicine residents, mainly because our former scheduling method for the formal curriculum resulted in numerous duplications and omissions, and sub-optimal resident/faculty satisfaction. Our map allowed us to proactively manage the logistics and relevance of formal curriculum delivery during AHD, while taking into account the specific requirements of teaching residents the non medical expert core competencies as mandated by the ACGME or RCPSC. The primary objective of this article is to describe the development of this real time curriculum map. The secondary objective is to assess the impact of implementing the map on formal curriculum planning and resident satisfaction.

## Methods

### Setting

The core internal medicine residency training program at the University of British Columbia (UBC) is a 3-year program with a 3-year cyclical formal curriculum. There were 72 residents in our residency program in 2003–2004 (26 in post graduate year (PGY)-1, 26 in PGY-2, 20 in PGY-3), 74 in 2004–2005 (26 PGY-1, 20 PGY-2, 28 PGY-3), and 81 in 2005–2006 (31 PGY-1, 31 PGY-2, 19 PGY-3). The bulk of the residency training experience involved clinical rotations in general internal medicine (clinical teaching units) and medical sub-specialties. Although each rotation had specific educational objectives, formal curriculum delivery was predominantly unstructured and determined by individual preceptors. The main structured teaching opportunity involved a weekly AHD program, which ran for 4 hours every Wednesday afternoon and was a mandatory component of the program. Of the weekly 4 hours, 3 hours had scheduled teaching activities (the focus of the curriculum map), with the remaining 1 hour for personal independent learning. AHD was protected time for residents and all services were expected to release them from ward and patient duties.

### Curriculum Map Development

In October 2004, we assembled the first 3-year cycle of the AHD curriculum map. We retrospectively used the 2003–2004 curricular data as baseline (which became year 1 of a 3-year cycle), and prospectively planned for the subsequent 2 years (2004–2005 and 2005–2006). The map was in the form of a matrix that comprised of 20 themes, which represented 16 specialties and 4 non medical expert themes (biomedical ethics, evidence based medicine, quality improvement, teaching skills). One hour of scheduled AHD time was allotted to each topic. Topics belonging to the same specialty/theme were grouped together in consecutive rows, and the hours of AHD coverage in each academic year were plotted in consecutive columns. All topics covered were cross-matched against the list of examination objectives from each year's ACP-ASIM in-training examination. The latter information was disseminated to all program directors after the annual examinations. The topics were colour coded to indicate those that were covered and examined, those covered but not examined, and those examined but never covered. The hours were also colour coded to indicate the category of core competencies covered, which paralleled those mandated by the national accreditation bodies. The map was updated monthly by the residency program secretary on an ongoing basis. A sample portion of the curriculum map is illustrated in the Appendix. We received approval from the UBC residency training committee to develop and implement the curriculum map and disseminate our experience.

Prior to the development of a curriculum map (baseline), AHD topics and speakers were determined by 2 of the 4 chief medical residents, without cross reference to what was previously taught and when it was taught. After implementation of the map, we assessed teaching needs on an ongoing basis. The topic, format (didactic and/or interactive) and speaker selection at AHD were determined by the AHD organizing committee, which comprised of 17 members (4 chief medical residents, 2 class representatives from each of the 3 years, and an additional 2 representatives from each class who expressed special interest in medical education, and chaired by the associate program director). The committee met every 4–6 weeks for about 90 minutes, during which members used the curriculum map to formulate a list of topics that fit into a series of predetermined AHD block themes. Members suggested potential speakers for these topics, with consideration of adequate and balanced representation based on geography (teaching or community hospitals) and expertise (clinical or research), and then extended personal invitations to the speakers. The information was collated by the chief medical residents and approved by the associate program director. On an ongoing basis, AHD topics were entered into the curriculum map by the program secretary.

### Curriculum Map Evaluation

Evaluation of the AHD curriculum map was conducted from 2 perspectives: formal curriculum planning and resident satisfaction. The impact of the map on formal curriculum planning was assessed by the percentage of AHD coverage that represented core competencies in internal medicine, the extent of topic duplication, the amount of didactic versus non-didactic teaching sessions, and the amount of teaching on non medical expert competencies as mandated by the national accreditation bodies. Resident satisfaction was evaluated using a structured, 5-item survey administered after each AHD for the period July 1, 2003 to June 30, 2006, which provided baseline data (2003–2004), and evaluation data in the first (2004–2005) and second (2005–2006) years after implementation of the curriculum map. Residents were asked to rate each survey item on a Likert scale from 1 (minimum satisfaction) to 4 (maximum satisfaction), with higher numeric scores representing better satisfaction.

Descriptive statistics (cumulative frequencies and relative percentages) were used to describe the content of the curriculum map. All scores from the satisfaction surveys were reported as mean ± SD. Post-implementation satisfaction scores were compared with baseline using 2-tailed, unpaired t tests, and differences were considered significant when p value < 0.05. All analyses were performed using SPSS 11.0 statistical software package (SPSS Inc. Chicago, IL).

## Results

The inaugural 3-year cycle of the AHD curriculum map included 283 scheduled hours. A total of 208 topics were covered, all of which (100%) represented core competencies in internal medicine.

Duplication of topics was minimal: most topics (163 or 78%) were covered once during the 3-year cycle. There were 30 topics (14%) that were covered twice in 3 years (e.g. general discussion on biomedical ethics, endocrine emergencies, thrombophilic conditions, HIV workshops, breast cancer, pulmonary function, etc.), and 11 topics (5%) were covered 3 times (e.g. acute coronary syndrome, diabetes mellitus, gastrointestinal bleeds, physical examination workshops, electrocardiography workshops, acid base disorder workshops, etc.) Two topics were covered 4 times in this formal curriculum cycle: sepsis/septic shock management, and the dermatologic manifestations of systemic disease. The topic on the principles of evidence based medicine was covered 5 times in 3 years.

The format of most AHD hours (241 or 85%) involved didactic activities, with the remaining 42 hours (15%) involved non-didactic teaching (such as physical examination workshops, case-based sessions, etc.). The latter increased substantially after implementation of the curriculum map, from 5 hours in 2003–2004, to 19 and 18 hours in 2004–2005 and 2005–2006 respectively.

The number of structured teaching hours in each of the 7 roles (core competencies) mandated by accreditation is summarized in Table 1. Of the scheduled AHD hours, most (220 hours or 78%) focused on competencies related to medical expertise (Table 1). The remaining hours covered non medical expert competencies in scholarly activities (16%), management skills (4%) and professional skills (2%). Of note, there was no specific AHD coverage of competencies on collaboration, communication and health advocacy in this formal curriculum cycle (Table 1). The proportion of AHD hours spent on non medical expert competencies increased over time: 19/90 (21%) in 2003–2004, 19/96 (20%) in 2004–2005, 23/97 (24%) in 2005–2006.

**Table 1:** Coverage of medical expert and non medical expert categories during academic half day classified by specialty/theme in a 3-year cycle of the formal curriculum map (2003 to 2006).

Specialty	Medical Expert	Collaborator	Communicator	Professional	Advocate	Scholar	Manager
Allergy and Immunology	5						
Biomedical Ethics				5			
Cardiology	22					6	
Critical Care	12						
Dermatology	8						
Endocrinology	21					3	
Evidence Based Medicine						18	
Gastroenterology	17					2	
General Internal Medicine	8			2		3	4
Geriatrics	7					1	
Hematology	20						
Infectious Diseases	17					2	
Maternal Fetal Medicine	9						
Medical Oncology	9						
Nephrology	18					2	
Neurology	15					1	
Quality Improvement							6
Respiratory	17					1	
Rheumatology	15					3	
Teaching Skills						4	
All specialties combined	220	0	0	7	0	46	10

The numbers represent hours of coverage in the formal curriculum. The 7 roles (core competencies) are based on accreditation requirements defined by the Royal College of Physicians and Surgeons of Canada (2).

Among the 283 scheduled AHD hours, 83 hours (29%) of which involved topics that were tested in the annual ACP-ASIM in-training examination. The percentage of AHD hours covering topics that were examined did not vary much: 28/90 (31%) in 2003–2004, 29/96 (30%) in 2004–2005, 23/97 (24%) in 2005–2006. The specialties/themes with the highest proportion of AHD hours covered and examined were geriatrics (75%), medical oncology (67%), gastroenterology (63%), and respiratory (50%) respectively (Table 2). There were 530 topics identified as examined but never covered at AHD, notably in general internal medicine (85 topics or 16%), infectious diseases (73 topics or 14%), hematology (53 topics or 10%) and rheumatology (48 topics or 9%).

**Table 2:** Specialty topic/theme coverage during academic half day in a 3-year cycle of curriculum map (2003 to 2006).

Specialty	Hours (%) of topics covered and examined within specialty	Hours (%) of topics covered but not examined within specialty	Total hours (%) of topics covered across all specialties	Number of topics examined but not covered within specialty
Allergy and Immunology	1 (20)	4 (80)	5 (2)	1
Biomedical Ethics	0 (0)	5 (100)	5 (2)	1
Cardiology	8 (29)	20 (71)	28 (10)	38
Critical Care	0 (0)	12 (100)	12 (4)	5
Dermatology	0 (0)	8 (100)	8 (3)	11
Endocrinology	9 (38)	15 (62)	24 (9)	27
Evidence Based Medicine	0 (0)	18 (100)	18 (6)	0
Gastroenterology	12 (63)	7 (37)	19 (7)	38
General Internal Medicine	4 (24)	13 (76)	17 (6)	85
Geriatrics	6 (75)	2 (25)	8 (3)	23
Hematology	4 (20)	16 (80)	20 (7)	53
Infectious Diseases	8 (42)	11 (58)	19 (7)	73
Maternal Fetal Medicine	0 (0)	9 (100)	9 (3)	2
Medical Oncology	6 (67)	3 (33)	9 (3)	22
Nephrology	9 (45)	11 (55)	20 (7)	31
Neurology	4 (25)	12 (75)	16 (6)	27
Quality Improvement	1 (17)	5 (83)	6 (2)	0
Respiratory	9 (50)	9 (50)	18 (6)	45
Rheumatology	2 (11)	16 (89)	18 (6)	48
Teaching Skills	0 (0)	4 (100)	4 (1)	0
All specialties combined	83 (29)	200 (71)	283 (100)	530

Examination refers to the ACP-ASIM in-training examination in the respective years.

While we mainly focussed on evaluating the process of creating a curriculum map, we also reviewed resident satisfaction surveys that were received for 255 of the total 283 hours (90% response rate) in the 3-year formal curriculum cycle (Table 3). The baseline satisfaction ratings prior to implementation of the curriculum map were high, ranging from 3.64 ± 0.21 to 3.78 ± 0.16 out of 4. After the first year of implementing the map, satisfaction ratings improved in all 5 survey items, and reached statistical significance for the items on evidence-based nature, preparedness of the speaker, presentation skills, and questions/discussion raised (Table 3). Interestingly, after the second year of implementation, satisfaction ratings trended back towards baseline values. Of note, the magnitude of changes over time remained small.

**Table 3:** Resident evaluation of academic half day before, during and after formal curriculum changes introduced based on curriculum map.

Evaluation Domains	Baseline (7/1/2003 – 6/30/2004) n = 79 hours	Year 1 post curriculum map (7/1/2004 – 6/30/2005) n = 89 hours	p value for difference between Year 1 and Baseline	Year 2 post curriculum map (7/1/2005 – 6/30/2006) n = 87 hours	p value for difference between Year 2 and Baseline
Topic	3.78 ± 0.16	3.82 ± 0.17	0.17	3.70 ± 0.22	0.01
Evidence-based	3.65 ± 0.25	3.76 ± 0.18	0.001	3.65 ± 0.26	0.99
Preparedness	3.77 ± 0.15	3.84 ± 0.14	0.002	3.73 ± 0.23	0.16
Presentation skills	3.72 ± 0.20	3.80 ± 0.16	0.005	3.68 ± 0.26	0.29
Questions/discussion	3.64 ± 0.21	3.75 ± 0.20	0.001	3.66 ± 0.28	0.73

All satisfaction scores are reported as mean ± SD. Possible score range 1 (minimum satisfaction) to 4 (maximum satisfaction), with higher numeric score representing better satisfaction.

## Discussion

We developed and implemented a cyclical curriculum map for our internal medicine residency program based on real time resident input. The map provided easy visualization of structured teaching activities that occurred at AHD, as well as an opportunity to continuously review the format (didactic versus non-didactic) and nature of teaching (medical versus non medical expert competencies). In the first 3-year cycle, all topics covered were relevant and represented core competencies, with minimum topic duplication. The curriculum map allowed any duplication to be reviewed and/or rationalized. Resident satisfaction remained high after implementing the map. These findings appear promising.

A curriculum map can identify potential gaps so that appropriate action can be taken [16]. For instance, only 29% of the scheduled hours covered topics that were actually tested in past ACP-ASIM examinations. While we recognized that our AHD should not merely cover competencies that were tested in the past, and not all competencies were testable in a multiple-choice format, we discovered a substantial number of topics that were examined but never formally covered at AHD, especially in general internal medicine, infectious diseases, hematology and rheumatology. These mirrored the same content areas where our resident performance in the ACP-ASIM examination was lower. The curriculum map may help to highlight deficiencies in topic coverage within these specialties for future educational planning.

Teaching gaps arise within the context of curricular changes, and the real time nature of the curriculum map permits ready incorporation of dynamic changes. For example, there was no specific AHD coverage of competencies on collaboration, communication and health advocacy in the first 3-year formal curriculum cycle, although we were mandated to teach these competencies by the accreditation bodies. These were relatively new areas in the formal curriculum, which required innovative and conscientious efforts to teach well. A curriculum map can help to unmask competencies that may have become hidden otherwise [17]. In the new cycle of curriculum map, we have now introduced new theme blocks to focus on competencies of collaboration, communication, health advocacy, as well as other medical content areas of palliative care and peri-operative medicine. Another example of change involved the introduction of more non-didactic, interactive sessions. The majority of our AHD activities involved didactic teaching using the classical pedagogic method of lectures, which is common in internal medicine [18]. The curriculum map made it logistically easier to track the gradual increase in non-didactic sessions. During the study period, non-didactic hours increased substantially after implementation of the curriculum map. Adult learners generally learn better with interaction, and it would be interesting to see this if the interactive, non-didactic sessions proved to be better rated than the didactic ones. Future studies with a larger sample size are warranted.

We realized that AHD could not be the only structured teaching opportunity to deliver a large internal medicine formal curriculum, and other venues such as noon-hour rounds and special retreats could be deployed to teach some competencies. We recognize omitting other educational experiences would affect the selection or distribution of topics in the AHD part of the curriculum. In the new formal curriculum cycle, we have expanded the curriculum map to include teaching at these venues. To help keep things simple and user friendly, we elected not to include topics covered during clinical rotations, although the latter was attempted in a recent study [19].

Resident satisfaction was high before and after implementing the curriculum map. The satisfaction ratings improved modestly after the initial year, suggesting residents viewed positively the changes in AHD as driven by the curriculum map. It is unclear why the same ratings returned to baseline 2 years later. Perhaps by year 2, some of the new residents were never exposed to the baseline AHD experience and therefore had no comparison. Other possible explanations include higher resident expectations after the initial changes imposed by the map, or the need for further acceptance and/or fine-tuning for the newly introduced non medical expert topics. Our observation can also be explained by the Hawthorne effect and/or regression to the mean.

Besides satisfaction scores, there are other interesting outcomes as we evaluate the impact of the curriculum map longitudinally. For instance, the score averages from public certification examinations can reflect clinical knowledge from our residents, although such confidential information is not readily accessible for the residency program. Future practice choices and career trajectories are also of interest.

Our study findings have some limitations. This is a single site formal curriculum and may not be generalizable to other institutions. We reported data from 1 formal curriculum cycle, and are therefore uncertain as to whether the same conclusions noted can be applied to future cycles. The changes in resident satisfaction ratings over time were small, probably due to the ceiling effect of the unvalidated survey. Future efforts to validate the survey will be helpful.

## Conclusion

In summary, we developed and implemented an internal medicine curriculum map based on real time resident input. Initial evaluation of this educational tool is promising. This has potential for broader implementation by residency programs as it highlights the spatial organization and interconnections between what is taught in the formal curriculum, how it is taught, and when it is taught, all within the context of teaching accreditation requirements in a finite period of training duration. The curriculum map requires ongoing needs assessment, and creates an opportunity of engaging residents to actively participate in their own education.

## Competing interests

The author(s) declare that they have no competing interests.

## Authors' contributions

RYW was the principal investigator of the study, developed and delivered the curriculum map, supervised the data collection and analysis, and was the principal author for the paper. JMR collaborated during the development of the curriculum map, reviewed and improved this paper. All authors read and approved the final manuscript.

## Appendix

**Table 4:** A sample portion (General Internal Medicine) of the UBC Department of Medicine Postgraduate Education Curriculum Map.

**Speciality and Topics**	**2003–2004**	**2004–2005**	**2005–2006**
**General Internal Medicine**			
Topics covered in Academic Half Day:			
**Eating Disorders**	1 (ME)		
Errors as a Learning Opportunity			1 (P)
Ethics of Symptom Management			1 (P)
Evidence Based Medicine: General Internal Medicine			1 (S)
Fever in the Returning Traveller			1 (ME)
**Hypertension (including Isolated Systolic Hypertension)**			1 (ME)
Insurance and Introduction to Practice Management			1 (M)
Optimization of Drug Therapy in Patients with Liver Disease		1 (ME)	
Palm Project in Medicine	2 (S)		
Peri-operative Medicine		1 (ME)	
Principles of Drug Therapy	1 (ME)		
Pros and Cons of Being a Community Internist		1 (M)	
**Secondary Hypertension, Laboratory Investigations of**		1 (ME)	
Things They Didn't Teach You in Medical School	1 (M)	1 (M)	
**Toxicology**		1 (ME)	
Topics not covered in Academic Half Day:			
Anti-obesity drugs			
Achilles tendonitis – from fluoroquinolone antibiotics			
Atrophic vaginitis			
Botulism			
Brain death			
Calculate incidence			
Candida albicans Infection			
Case control study			
Cellulitis			
Chlamydia trachomatis infection			
Chronic catheter-assisted urinary drainage			
Chronic recurrent sinusitis			
Chronic venous insufficiency			
Complement cascade			
Compression of the lateral femoral cutaneous nerve			
Contact dermatitis			
Dog bite			
Down's syndrome (risk for atlanto-axial instability)			
Dupytren's contracture			
End-of-life issues			
Epidural abscess			
Epistaxis			
Erythema multiforme			
Fat-soluble vitamins			
Garlic, ginseng and gingko biloba			
Genetic Diseases			
Glaucoma			
Heat stroke			
Hereditary angioedema			
Hidradenitis suppurativa			
Hyperthermia			
Hypophosphatemia			
Hypothermia			
Innocent cardiac flow murmur			
Ischemic encephalopathy			
Isopropyl alcohol ingestion			
Klinefelter's syndrome			
Lactose intolerance			
Lateral epicondylitis			
Likelihood ratios			
Low back pain			
LSD			
Male adolescent with gynecomastia			
Malignant external otitis			
Marked obesity			
Meniere's disease			
Meningitis			
Methanol ingestion			
Morton's neuroma			
Myoclonus			
Neurofibromatosis			
Number needed to treat			
Obstructive sleep apnea			
Palpitations			
Patient controlled analgesia			
Post-test probability			
Post-traumatic stress disorder			
Prevalence			
Preventive medicine			
Prostrate disease			
Psoriasis			
Renal stones			
Restless legs syndrome			
Rhabdomyolysis			
Rhinocerebral mucormycosis			
Risk reduction, absolute – number needed to treat			
Rotator cuff tendonitis			
Scurvy			
Secondary syphilis			
Serotonin syndrome			
Serum alkaline phosphatase elevation			
Sexually active homosexual man			
Sinusitis as a cause of halitosis			
Subconjunctival Hemorrhage			
Splenectomy and medical issues			
Symptomatic carotid artery stenosis			
Symptomatic urinary tract infections			
Systolic heart failure			
Tension headache			
Tinea pedis and lower extremity cellulitis			
Traveller			
Ulnar neuropathy			
Uncomplicated low back pain			

Topics are listed in consecutive rows, with the corresponding hours of academic half day (AHD) coverage in columns. All topics are cross-matched against examination objectives from each year's ACP-ASIM in-training examination. The topics are colour coded to indicate those covered and examined (bolded black font), those covered but not examined (non-bolded black font), and those examined but never covered (red font in the original map). The AHD hours are also colour coded to indicate the category of core competencies covered under the CanMEDS 2005 framework, including medical expert (ME, or white boxes in the original map), manager (M, or gold boxes), scholar (S, or rose boxes), and professional skills (P, or grey boxes). In this particular sample map, core competencies in health advocacy (green boxes), communication (yellow boxes) and collaboration skills (orange boxes) have not been covered in the formal curriculum cycle.

## Pre-publication history

The pre-publication history for this paper can be accessed here:


